# A Case of Curative Surgery after Effective Chemotherapy for Gastric Adenocarcinoma with Enteroblastic Differentiation Accompanied by Synchronous Multiple Liver Metastases

**DOI:** 10.70352/scrj.cr.25-0205

**Published:** 2025-07-09

**Authors:** Shuhei Yamada, Toshiki Wakabayashi, Isao Kikuchi, Michinobu Umakoshi, Masato Sageshima, Tsutomu Sato, Junichi Arita

**Affiliations:** 1 Department of Gastroenterological Surgery, Akita City Hospital, Akita, Akita, Japan; 2 Department of Gastroenterological Surgery, Akita University Graduate School of Medicine, Akita, Akita, Japan; 3 Department of Pathology, Akita City Hospital, Akita, Akita, Japan

**Keywords:** alpha-fetoprotein, gastric cancer, trastuzumab, preoperative chemotherapy, conversion surgery, adenocarcinoma with enteroblastic differentiation

## Abstract

**INTRODUCTION:**

Gastric adenocarcinoma with enteroblastic differentiation (GAED) is associated with a poor prognosis because of high rates of liver and lymph node metastases. While systemic chemotherapy is the standard treatment for gastric cancer (GC) with liver metastases, several studies suggest that hepatectomy, when combined with multimodal treatment, may provide a survival benefit. However, the role of surgical resection for GAED with liver metastases remains controversial.

**CASE PRESENTATION:**

A 71-year-old man presented with abdominal pain and nausea. Endoscopy revealed a type 2 tumor at the greater curvature of the gastric body. Contrast-enhanced computed tomography showed thickening and enhancement of the gastric wall, bulky lymph node metastases, and bilobar hepatic lesions, with the largest tumor measuring 60 mm in diameter. Histological examination of the stomach and liver tumors revealed adenocarcinoma composed of cuboidal or columnar cells resembling a primitive intestine-like structure with clear cells. Immunostaining showed heterogeneous cytoplasmic positivity for alpha-fetoprotein and spalt-like protein 4, leading to a diagnosis of GAED with liver metastases. Because the tumor was positive for human epidermal growth factor receptor 2 (HER2), chemotherapy with capecitabine, cisplatin, and trastuzumab was administered. After six cycles, the tumors had significantly decreased in size, and curative-intent surgery was performed, including distal gastrectomy, left lateral sectionectomy, and partial hepatectomy, successfully eradicating all five liver metastases. Histological examination of the liver metastases revealed extensive necrosis and fibrosis with no viable tumor cells. Adjuvant chemotherapy with the same regimen was continued for 1 year. At the time of this writing, the patient had remained recurrence-free for more than 2 years postoperatively.

**CONCLUSIONS:**

We report a rare case of GAED with multiple liver metastases successfully treated with aggressive surgical resection following systemic chemotherapy. Trastuzumab-based chemotherapy may be a viable treatment option for HER2-overexpressing GAED. In addition, radical surgery for GAED with liver metastases might prolong the survival if the chemotherapeutic regimen was effective.

## Abbreviations

AFP
: alpha-fetoprotein
AFPGC
: alpha-fetoprotein-producing gastric cancer
GAED
: gastric adenocarcinoma with enteroblastic differentiation
GC
: gastric cancer
HAC
: hepatoid adenocarcinoma
HER2
: human epidermal growth factor receptor 2
PD-L1
: programmed cell death ligand 1
SALL4
: spalt-like protein 4

## INTRODUCTION

GAED has been recognized as a variant of AFPGC. Previous studies have reported that GAED is associated with a poor prognosis because of its high incidence of liver metastases.^1,2)^ The standard treatment for GC with multiple liver metastases is chemotherapy. With chemotherapy alone, the median survival for GC with distant metastases is reportedly 10–15 months.^3–6)^ However, because of its rarity, it remains unclear whether chemotherapy for GAED is as effective as for other major histologic subtypes of GC.^7–9)^ New anticancer agents, including molecular-targeted drugs, have recently been developed. The ToGA trial demonstrated that a recombinant humanized monoclonal antibody targeting HER2 significantly prolonged overall survival in patients with HER2-positive GC.^10)^ Given that HER2 positivity tends to be higher in GAED than in conventional GC, patients with GAED may be good candidates for anti-HER2 therapy.^11–13)^ Additionally, previous studies have reported that surgical resection for GC with liver metastases can improve survival rates in select patients.^14–17)^

We herein report a case of HER2-positive GAED with synchronous multiple liver metastases, in which preoperative chemotherapy, including trastuzumab, was effective. We successfully performed surgical resection, and the patient was clinically well without any recurrence at the time of this writing.

## CASE PRESENTATION

**Fig. 1:**
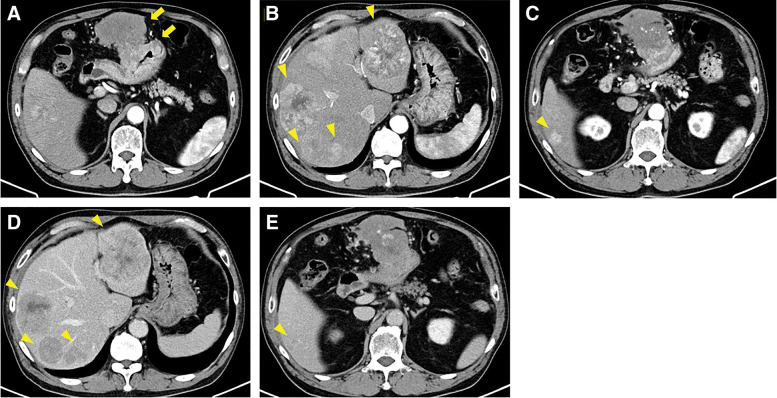
(**A**) Contrast-enhanced computed tomography showing thickening and enhancement of the gastric body wall with bulky lymph node swelling (arrows). (**B**–**E**) Five bilateral hepatic lesions, with the largest measuring 60 mm, exhibiting central necrosis and heterogeneous enhancement. All liver tumors demonstrated arterial hyperattenuation and washout on dynamic computed tomography (**B** and **C**, early phase; **D** and **E**, late phase; arrowheads).

A 71-year-old man presented with abdominal pain and nausea. His medical history included systemic hypertension, angina pectoris, and gastric ulcer. Laboratory data, including liver enzyme levels, were all within the normal range (**Table 1**). Tumor marker levels were as follows: AFP, 552 ng/mL (normal: <10 ng/mL); protein induced by vitamin K absence-II, 23 mAU/mL (normal: <40 mAU/mL); carcinoembryonic antigen, 23 ng/mL (normal: <5.0 ng/mL); and carbohydrate antigen 19-9, 185 U/mL (normal: <37 U/mL). Contrast-enhanced computed tomography revealed thickening and enhancement of the gastric body wall, bulky lymph node swelling, and five bilobar hepatic lesions, the largest measuring 60 mm with central necrosis and heterogeneous enhancement. All liver tumors exhibited hyperattenuation in the arterial phase and washout in the venous phase. The liver surface was smooth, the liver edge was sharp, and no epigastric venous dilatation was observed (**Fig. 1**). Upper gastrointestinal endoscopy revealed a 50- × 40-mm type 2 tumor at the greater curvature of the gastric body.

**Table 1:** Laboratory data

RBC	4.35 10^6^/μL	PT	116 %
Hb	13.4 g/dL	APTT	27.2
WBC	5.5 10^3^/μL	Glucose	110 mg/dL
Plt	21.3 10^4^/μL	HbA1c	5.3 %
T.bil	0.54 mg/dL		
D.bil	0.12 mg/dL	HBsAg	0 IU/mL
AST	41 U/L	HBcAg	0 IU/mL
ALT	16 U/L	HCVAb	0 C.O.I
ALP	132 U/L		
LDH	543 U/L		
GTP	251 U/L		
BUN	13.8 mg/dL		
CRE	1.12 mg/dL		
Na	138 mmol/L		
K	4.6 mmol/L		
CL	106 mmol/L		
TP	6.1 g/dL		
ALB	3.7 g/dL		
CRP	0.87 mg/dL		

**Fig. 2:**
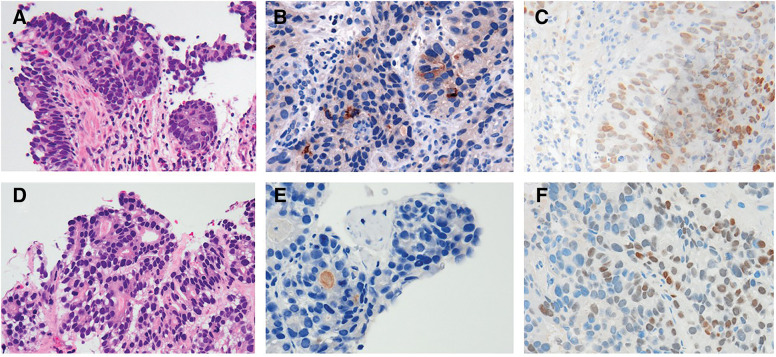
Histological features of the biopsied gastric and liver specimens. (**A**) Gastric tissue showing poorly differentiated adenocarcinoma with a tubular growth pattern and clear cells. In addition, tumor cells composed of cuboidal or columnar cells resembling a primitive intestine-like structure with clear cells (H&E, 200×). (**B**) Gastric tumor cells moderately stained for AFP (200×). (**C**) Gastric tumor cells diffusely stained for SALL4 (200×). (**D**) The liver biopsy specimens closely resemble the gastric specimens in morphology (H&E, 200×). (**E**) Liver tumor cells faintly stained for AFP (200×). (**F**) Liver tumor cells diffusely stained for SALL4 (200×). AFP, alpha-fetoprotein; H&E, hematoxylin and eosin; SALL4, spalt-like protein 4

An endoscopic biopsy confirmed a histological diagnosis of poorly differentiated adenocarcinoma with HER2-positive status. Because of the hypervascularity of the liver tumors, hepatocellular carcinoma and liver metastases from GC were considered as differential diagnoses. A percutaneous needle biopsy was subsequently performed, revealing a histological diagnosis of tubular adenocarcinoma. Immunostaining of the liver specimen showed faint cytoplasmic positivity for AFP. Additionally, the tumor cells consisted of cuboidal or columnar cells resembling a primitive intestine-like structure, with periodic acid-Schiff-positive clear cells in some areas. The tumor cells also demonstrated heterogeneous positivity for SALL4 by immunostaining. Similar histopathological features were also identified in a gastric specimen. SALL4 positivity might be similar between primary and metastatic lesions but AFP of primary lesion was much more than that of metastatic lesions (**Fig. 2**). Based on these findings, the hepatic lesions were diagnosed as liver metastases of GAED, a variant type of AFPGC.

We started chemotherapy with capecitabine/cisplatin plus trastuzumab. We followed the regimen used for major histologic subtypes of GC because no standardized chemotherapy protocol has been established for GAED due to its rarity.

**Fig. 3:**
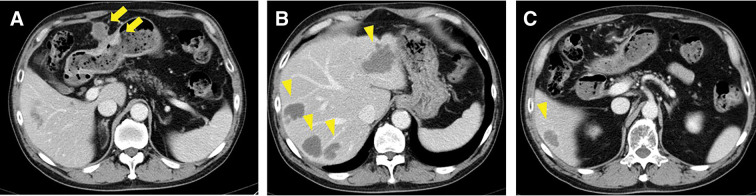
Tumor response after six courses of trastuzumab-containing chemotherapy. (**A**) Bulky lymph node swelling decreased in size, and gastric wall thickening improved (arrows). (**B**, **C**) Liver metastases shrank and demonstrated morphological changes; the largest hepatic tumor in the left lateral segment shrank to 46 × 35 mm, representing 59% of the original area (arrowheads).

**Fig. 4:**
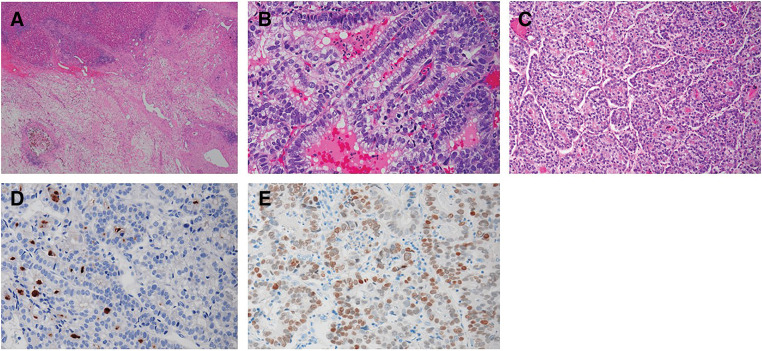
Histopathological features of the resected specimens. (**A**) Almost all liver tumors were replaced by fibrotic scar tissue, with no viable tumor cells observed (H&E, 20×). (**B**) The gastric tumor displayed tubular, poorly differentiated adenocarcinoma with clear cells, composed of cuboidal or columnar cells resembling a primitive intestine-like structure (H&E, 50×). (**C**) The hepatoid area, resembling hepatocellular carcinoma, was arranged in a nested pattern (H&E, 100×). (**D**) Tumor cells stained positively for AFP (100×). (**E**) Tumor cells diffusely stained for SALL4 (100×). AFP, alpha-fetoprotein; H&E, hematoxylin and eosin; SALL4, spalt-like protein 4

After six cycles of chemotherapy, the liver metastases markedly decreased in size and exhibited morphological changes; the largest hepatic tumor shrank to 46 × 35 mm (59% of its original area) and became homogeneous with low attenuation, as shown in **Fig. 3**. No additional metastatic tumors were detected in imaging studies, and the chemotherapy response was classified as a partial response according to version 1.1 of the Response Evaluation Criteria in Solid Tumors. All tumor markers returned to normal levels, prompting the decision for curative-intent surgery as adjuvant therapy. Six weeks after chemotherapy, the patient underwent distal gastrectomy with D2 lymph node dissection, Billroth II reconstruction, left lateral sectionectomy, and two partial hepatectomies. The surgery lasted 8 hours and 21 minutes, with blood loss of 652 mL, and no blood transfusion was required. Histopathological examination revealed that nearly all liver tumors were replaced by fibrous and necrotic tissue, with no viable tumor cells remaining (**Fig. 4A**), and the chemotherapy efficacy for hepatic specimens was graded as Evans grade 3. By contrast, the gastric tumor, measuring 45 × 35 mm on the posterior wall, contained viable malignant cells and had infiltrated the subserosal layer without lymph node metastasis. A previously suspected metastatic lymph node showed cystic changes due to chemotherapy. Histologically, the tumor cells were tubular and poorly differentiated adenocarcinoma with clear cells, composed of cuboidal or columnar cells resembling a primitive intestine-like structure (**Fig. 4B**). Additionally, hepatoid areas, mimicking hepatocellular carcinoma with a nested pattern, were present in some regions (**Fig. 4C**). While residual tumor cells were observed in the gastric tumor, contrary to the liver tumors, both AFP and SALL4 were positive (**Fig. 4D** and **4E**). The histological findings of the resected gastric tumor were consistent with those of the preoperatively biopsied liver tumors, leading to a final diagnosis of GAED and HAC. According to the Evans grading system, the chemotherapy efficacy for the gastric tumor was classified as grade 1b.

The postoperative course was uneventful, and the patient was discharged on postoperative day 14. The same chemotherapy regimen, including trastuzumab, was continued as adjuvant therapy and completed after 1 year. At the time of this writing (>3 years after surgery), the patient remained in good health with no signs of tumor recurrence.

## DISCUSSION

AFPGC is a rare subtype of GC, accounting for 1%–7% of cases,^18,19)^ with GAED recognized as a variant of AFPGC. In this case, we performed radical resection for AFPGC following chemotherapy. While the standard treatment for GC with multiple liver metastases is chemotherapy, surgical resection has been shown to improve survival rates in select patients.^14–17)^ Previous studies have identified the number and size of hepatic tumors as poor prognostic factors after hepatectomy for GC.^15,20–25)^ Kinoshita et al.^15)^ reported prognostic factors for resectable GC liver metastases, suggesting that serosal invasion of the primary tumor, the presence of ≥3 liver metastases, and a largest hepatic tumor ≥5 cm are associated with poor overall survival. They emphasized that non-surgical treatment should be considered when any of these three factors are present at diagnosis.^15)^ In this case, the patient had two of these poor prognostic factors—five liver metastases and a maximum tumor diameter exceeding 5 cm. Accordingly, we initially selected chemotherapy with an anti-HER2 drug, opting for subsequent conversion surgery only after confirming a significant reduction in liver metastases. However, the indication for conversion surgery in patients with liver metastases from GC has yet to be clearly defined.^14–17)^ Although positron emission tomography scan was not performed in the present case, a comparison of the images between before and after chemotherapy might support the validity of conversion surgery.

Previous reports have shown that AFPGC is associated with a high incidence of lymphatic and venous invasion, synchronous and metachronous liver metastases, and poor pTNM stage.^26–29)^ Adachi et al. analyzed 270 cases of AFPGC and reported that curative resection improved survival rates; however, nearly all deaths were attributed to liver metastases, with a median survival of only 15 months.^26)^ In previous studies, six cases of conversion surgery after chemotherapy for AFPGC have been reported, all leading to favorable prognoses (**Table 2**).^30–35)^ The histological subtype was HAC in four cases, GAED in one case, and unknown in the remaining case. Chemotherapy regimens included cisplatin or oxaliplatin in five cases, while nivolumab monotherapy was used in one case for metachronous liver metastases. In three of the six cases, curative resection was performed for liver metastases, resulting in a median survival of 25 months.^30,34,35)^ In the present case, bilobar liver metastases were observed, a condition not previously reported; however, the patient had remained alive and recurrence-free for 3 years at the time of this writing. This outcome aligns with previous reports, suggesting that long-term survival may be achievable.

**Table 2:** Review of conversion surgery of AFPGC

Report	Histological type	Sex	Age	Metastases/Invasion	Preoperative chemotherapy	Surgical procedure	Clinical course	Reported survival
Ye et al. 2013^30)^	HAC	Male	58	Multiple liver metastases tumor thrombosis in the portal vein	4 cycles of epirubicin, oxaliplatin, and fluorouracil 6 cycles of capecitabine and oxaliplatin	Distal gastrectomy with left lateral sectionectomy	No recurrence	20 months (alive)
Becq et al. 2015^31)^	HAC	Male	69	Infiltration into left liver and splenic hilum paraaortic lymph node metastases	4 cycles of epirubicin, oxaliplatin, and capecitabine	Total gastrectomy with splenectomy, left hemihepatectomy, and partial phrenicotomy	No recurrence	31 months (alive)
Nakao et al. 2016^32)^	Unknown	Male	63	Tumor thrombosis in the portal vein	3 cycles of S-1 and cisplatin	Total gastrectomy	No recurrence	48 months (alive)
Shen et al. 2016^33)^	HAC	Male	70	Infiltration into left liver	2 cycles of capecitabine and oxaliplatin	Resection of the stomach and external lobe of left liver	No recurrence	7 months (alive)
Simmet et al. 2018^34)^	HAC	Male	64	Three lesions of liver metastases	6 cycles of cisplatin and etoposide	Gastrectomy and right hemihepatectomy	No recurrence	More than 9 years (alive)
Jun et al. 2023^35)^	GAED	Female	69	Two lesions of liver metastases	18 cycle of nivolmab	Partial hepatectomy	No recurrence	25 months (alive)
Present case	HAC + GAED	Male	71	Six lesions of liver metastases	6 cycles of cisplatin, capecitabine, and trastuzumab	Distal gastrectomy with left lateral sectionectomy and partial hepatectomy	No recurrence	28 months (alive)

GAED, gastric adenocarcinoma with enteroblastic differentiation; HAC, hepatoid adenocarcinoma

The standard diagnosis of AFPGC, including GAED, is based on elevated serum AFP levels combined with specific histological findings. In GAED, solid areas consist of large cells with hyperchromatic nuclei and abundant clear cytoplasm containing intracytoplasmic periodic acid-Schiff-positive droplets, resembling a primitive intestine-like structure.^2,36)^ Additionally, the importance of immunological positivity for glypican 3 and SALL4 as enteroblastic markers has been increasingly recognized.^7,37,38)^ In the present case, SALL4 was diffusely positive. HAC, another subtype of AFPGC, is characterized by hepatoid features arranged in a tubular or solid nested growth pattern with large polygonal hepatoid-like neoplastic cells.^39,40)^ However, this area also exhibited clear hepatoid features, and both GAED and HAC are representative subtypes of GC with clear cell morphology.^7,36,41)^ Because of their rarity and histologic overlap, differentiating GAED from HAC can be challenging for pathologists. Previous reports suggest that more than half of AFPGC cases present as mixed types, incorporating HAC, GAED, and conventional adenocarcinoma.^27,42)^ In the present case, histological diagnosis was difficult because features of both GAED and HAC were observed: a primitive intestine-like structure and a nested growth pattern with large polygonal hepatoid-like neoplastic cells. However, the absence of sinusoid-like vascular channels in the solid pattern served as a distinguishing characteristic from HAC, making a diagnosis of GAED more likely than HAC.

**Fig. 5:**
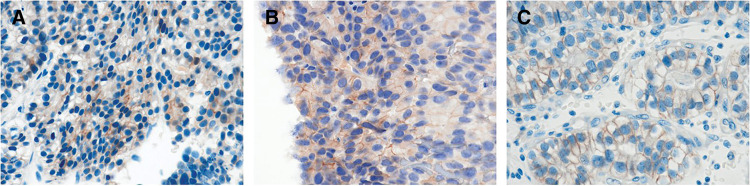
HER2 positivity of primary and metastatic lesions both before and after treatment. (**A**) Gastric tumor cells before treatment moderately stained positively for HER2 (100×). (**B**) Liver tumor cells before treatment strongly stained positively for HER2 (100×). (**C**) Gastric tumor cells after treatment moderately stained positively for HER2. HER2 expression of primary lesion before treatment was not as much as that after treatment (100×). HER2, human epidermal growth factor 2

The optimal chemotherapy regimen for GAED has not yet been established. Previous studies have reported frequent overexpression of HER2 and PD-L1 in GAED and HAC.^11,13,43–45)^ Trastuzumab, an anti-HER2 drug, has been shown to be effective in patients with advanced HER2-positive GC, as demonstrated in the large-scale phase 3 international ToGA clinical trial.^10)^ Fujimoto et al. reported HER2 positivity rates of 31% in HAC and 42% in GAED,^12)^ both higher than the 22.1% observed in conventional GC.^10)^ These findings suggest that patients with GAED and HAC may be strong candidates for anti-HER2 therapy. In the present case, chemotherapy including trastuzumab was effective, resulting in histological complete remission in the resected liver specimens. However, the primary gastric lesion remained predominantly viable. In the present case, HER2 expression of liver specimens was more clustered than that of primary lesions (**Fig. 5A** and **5B**). In addition, HER2 expression of primary lesions before treatment was not as much as that after treatment (**Fig. 5A** and **5C**). This discrepancy may be attributable to the reported heterogeneity in HER2 amplification rates between primary and metastatic gastric cancer lesions, which could influence differential responses to trastuzumab-based chemotherapy.^46–48)^

## CONCLUSIONS

We encountered a rare case of GAED with multiple liver metastases successfully treated with systemic therapy followed by curative-intent surgical resection. Although GAED is associated with a poor prognosis, preoperative anti-HER2 therapy aimed at conversion surgery may serve as a potential treatment option.

## ACKNOWLEDGMENTS

We thank Angela Morben, DVM, ELS, from Edanz (https://jp.edanz.com/ac), for editing a draft of this manuscript.

## DECLARATIONS

### Funding

The authors declare that they have not received any funding for this report.

### Authors’ contribution

SY drafted the manuscript.

TW, IK, and TS treated the patient.

MU and MS diagnosed this case based on pathological findings.

JA contributed suggestions and critiqued the manuscript.

All authors have read and approved the manuscript.

### Availability of data and materials

Not applicable.

### Ethics approval and consent to participate

This work does not require ethical considerations or approval. Informed consent to participate in this study was obtained from the patient.

### Consent for publication

Informed consent was obtained from the patient for this publication.

### Competing interests

The authors declare no conflicts of interest.

### Use of artificial intelligence tools

We did not use any AI tools.
